# Exploring Emergency Healthcare for Women Experiencing Intimate Partner Violence With Comorbid Psychological Distress

**DOI:** 10.1111/inm.70050

**Published:** 2025-04-15

**Authors:** Shannon Dhollande, Diksha Sapkota, Silke Meyer, Karen‐Ann Clarke, Maria Atiénzar‐Prieto

**Affiliations:** ^1^ CQUniversity Brisbane Australia; ^2^ Griffith University Nathan Australia; ^3^ University of the Sunshine Coast Sippy Downs Australia

**Keywords:** domestic abuse, domestic and family violence, emergency, emergency department, intimate partner violence, mental health, nursing

## Abstract

Intimate partner violence (IPV) may cause significant mental and physical health deterioration. This gendered violence often results in victims seeking support from emergency healthcare providers. Yet very little is known about the care provided to these patients. The objective of this research was to provide a descriptive analysis of care provision within the emergency setting to women experiencing IPV with concurrent psychological distress. A retrospective chart review was undertaken. This included screening 300 patient charts with inclusion/exclusion criteria to arrive at a final sample size of 32 patient charts comprising 43 presentations from 2020 to 2022. Whilst IPV is being recognised within emergency healthcare, clinician responses suggest a pathologising of symptoms associated with IPV victimisation and a focus on physiological care. Pathways in place to promote patient safety and outpatient services were rarely utilised. Furthermore, paramedics were seen to have removed autonomy from patients using Public Health Legislation, potentially causing secondary re‐victimisation. There are several ways in which healthcare clinicians can improve their care to patients experiencing IPV. Education surrounding healthcare roles and responsibilities and family violence legislation may be central to improving service provision. Knowledge of and referral to appropriate outpatient support services may also be a method by which to address ongoing health and safety concerns.

## 1 Introduction

Intimate partner violence (IPV) is a common form of violence that includes different forms of behaviour (e.g., emotional, sexual, physical or financial abuse or violence) perpetrated by a current or former partner (World Health Organisation [WHO] 2004a). IPV is a significant public health problem that affects one in four women (27%) in Australia (Australian Bureau of Statistics 2024), and one in three women worldwide (33%, WHO 2024a, 2024b). There is a significant overlap between IPV and mental illness, with IPV victims having an increased risk of anxiety, depression, posttraumatic stress disorder, self‐harming behaviours and suicide compared to women without IPV victimisation experiences (Dekel et al. 2020; Potter et al. 2021; Rasmussen et al. 2023; Sapkota et al. 2021). IPV victims also report higher rates of engagement with risky health behaviours (e.g., alcohol use, harmful substance use and risky sexual practices) (Potter et al. 2021; Stubbs and Szoeke 2022). Earlier research has demonstrated the increased risk of repeated victimisation and worsening mental health among women who have experienced IPV (Cougle et al. 2009; Graham‐Kevan et al. 2015). Australia's latest National Plan to End Violence Against Women and Children 2022–2032 stipulates the need for holistic service system responses that ensure victim survivors' access to support and recovery beyond immediate crisis responses (Department of Social Services 2024).

Victims of IPV are among the most common groups accessing emergency health services for injury and noninjury‐related health concerns (NSW Ministry of Health 2024). Mental health concerns are among the most common reasons for emergency department (ED) presentations among IPV victims (Ghafournia and Healey 2022). For example, studies from Australia have documented that 36% of women presenting to EDs have experienced IPV in the last 18 months, and 47%–61% of women presenting to ED for mental health issues report experiences of IPV (Leckning et al. 2020; Rasmussen et al. 2021). Similar findings are noted in studies conducted internationally (Sprague et al. 2014). Therefore, ED clinicians are in a unique position to identify and provide support to victims of IPV. However, in most cases, clinical symptoms of mental illness or physical injuries are the predominant focus of treatment rather than the screening and identification of IPV (Gillespie et al. 2023). Although evidence supports the strong link between trauma and mental health symptoms, IPV‐related trauma is not routinely inquired about by ED clinicians (Hegarty et al. 2022), who may not consider that identifying and responding to IPV falls within their clinical role or who may not have enough time or necessary practice skills to accurately recognise and manage IPV (Gillespie et al. 2023). Most victims of IPV are also reluctant to disclose their IPV experiences, including when specifically asked when receiving care. However, there is substantial evidence that routine screening increases disclosure rates (cf. Heron and Eisma 2021). In the absence of such screening, the majority of IPV victims are likely to remain unidentified or inadequately supported in the day‐to‐day practices of EDs (Hegarty et al. 2022; Hinsliff‐Smith and McGarry 2017).

This failure to adequately identify IPV in EDs and recognise its contribution to adverse mental health outcomes among victim‐survivors may lead to the pathologisation of common responses to IPV victimisation, such as emotional distress, anxiety and other PTSD symptoms. This increases the risk of misdiagnosis and an absence of holistic responses that address mental health presentations and their contributing factors (Gillespie et al. 2023). Mental health presentation results in a lack of IPV‐related safety planning and support mechanisms and may thus increase the risk of re‐victimisation. This poses a missed opportunity for the reduction of fatalities and associated co‐morbidities, as evidenced by repeated visits to EDs and other medical services by victims (Hinsliff‐Smith and McGarry 2017).

Given the significant bidirectional relationship between mental illness and IPV, it is unlikely that the mental health issues will be resolved without addressing the concurrent, ongoing experiences of IPV (Humphreys et al. 2022). Whilst there is increasing national and international recognition of the importance of routine screening for IPV in ED and mental health care settings (García‐Moreno et al. 2021; Heron and Eisma 2021), there is currently no required routine screening for IPV undertaken by ED clinicians in Australia, and international research evidence reveals that routine screening implementation in ED settings remains consistently lower compared to other healthcare settings (Miller et al. 2021). Additionally, research supports the need for a holistic approach to the problem, emphasising individualised, trauma‐informed support to victim‐survivors of IPV (Paphitis et al. 2022). However, little is known about the care provisions available to victim survivors of IPV with concurrent mental health concerns once their history of victimisation is disclosed in the ED. This study contributes to filling an important gap in violence research by providing evidence on healthcare provisions for IPV victims presenting to the ED with concurrent mental health concerns.

## 2 Aim

The objective of this study was to explore care provisions, including clinical and psychosocial care, within an ED for women experiencing IPV with concurrent mental health concerns.

## 3 Methods

### 3.1 Study Setting

This project was undertaken in the ED of a regional hospital within Australia. This hospital services a large low socio‐economic population with educational attainment and employment rates below the state's average. In the jurisdiction where the study was conducted, healthcare clinicians are mandated reporters, required to report abuse of minors and the elderly. Healthcare clinicians in this region are not mandated to report IPV experienced by women over the age of 18 with cognitive capacity. Though there are policies and procedures in place to facilitate information sharing between support services, the police and/or with the High‐Risk Team operating in the study location with or without patient consent where cases meet a certain safety risk threshold (Domestic and Family Violence Protection Act 2012 (Qld) Part 5A (Austrl)).

### 3.2 Design

The researchers conducted a retrospective patient chart review. The retrospective patient chart review included reviewing all documentation generated by the patient's presentation including but not limited to written and electronic records, radiography, pathology and mental health records. The researchers explored not only what care tasks were undertaken but also how these were documented.

### 3.3 Sample and Data Screening

Due to the complexity of the way that documentation captures pertinent information, a three‐step approach of identifying eligible patients was employed. In step 1, three hundred (*N* = 300) patient charts were randomly sourced from the hospital's Emergency Department Information System (EDIS). The initial inclusion criteria for these charts were that the patient was female, at least 18 years old, presented to the ED between 1 January 2020 and 1 January 2022 and received a triage referral to mental health services within the hospital as part of this ED presentation.

In step 2, the entire research team of highly experienced academics and clinicians with experience in both IPV, mental healthcare and ED care met as a group and reviewed the first 50 charts to establish common language used by clinicians to identify IPV within the patient's current presentation. In this study, current IPV was defined as explicit terminology identified within the current ED presentation and which contributed to the patient's need for medical aid. Key terms included *physical abuse, punched, hit, pushed, strangled, choked, domestic violence* and *verbal abuse*. Charts which only described historical IPV or childhood abuse were excluded.

In step 3, the research group met on three separate occasions to screen the remaining 250 charts. At least two of the four members needed to be present for this screening, and consensus was reached using the previously established inclusion criteria. These charts were also reviewed to ensure the completeness of their documentation. Charts with missing data records were removed, as important information related to their mental or physical health care may have been absent. This resulted in a final sample of 32 patient charts, totalling 43 presentations within the stated period, all of which included clearly identifiable and current IPV.

### 3.4 Data Analysis

Data relating to the identified 43 presentations were manually extracted from the patient charts. A data extraction table was employed to extract data and then to import this data into statistical software SPSS version 28.0. The data extraction table was developed iteratively. Data extraction was completed by two members of the research team. Categories and labels were discussed and defined through discussion with various perspectives considered from at least three members of the interdisciplinary team. Statistical analysis included descriptives such as means, medians, percentages and frequencies.

## 4 Results

Patients' age ranged from 18 to 56 years. Patients were primarily born within Australia (*n* = 23, 71.9%) and 12.5% (*n* = 4) identified as Aboriginal Australians. At the time of their presentation, one patient was pregnant. When describing the violence they experienced, 48.8% (*n* = 21/43) of patients stated within their presentation that they were experiencing active IPV by their current partner, whereas 51.2% (*n* = 22/43) were experiencing IPV by an ex‐partner. Additionally, within the 43 emergency presentations, 53.3% (*n* = 23/43) documented that at the time of presentation, the women cared for children. Thursday (*n* = 9, 20.9%), Friday (*n* = 8, 18.6%) and Saturday (*n* = 7, 16.3%) were the most prevalent days in which IPV incidents occurred requiring ED care. To assess the level of clinical urgency (based on physiological observations) the triage scores for each presentation were analysed. In 90% (*n* = 39/43) of presentations, patients were triaged as requiring care within 30 mins, indicating a need for prompt intervention (Australasian Collage of Emergency Medicine [AECM], n.d.).

## 5 Patient Safety

In 60.5% (*n* = 26/43) of presentations, the patient was brought into the ED by an ambulance. In 80.7% (*n* = 21/26) of these transfers, Public Health Legislation in the form of an emergency examination authority [EEA] was used to facilitate the transfer to the hospital. Upon examination of the documented EEAs and corresponding paramedic notes, the legislative requirements for the initiation of an EEA were largely not substantiated. The legislative requirements include that the patient poses an immediate risk of harm to themselves or others, that they require urgent healthcare, and that they lack the cognitive capacity to make decisions regarding their bodily autonomy.

Within 58.1% (*n* = 25) of presentations there was no documentation surrounding whether there was a Domestic Violence Order (also known as apprehended violence orders) in place, nor any documented discussion on the potential need for one (Table 1). This apparent lack of attention to the patients' ongoing safety was further evidenced by the absence of documented safety planning undertaken with patients by different clinicians. Safety planning was absent in 83.7% (*n* = 36) of documented patient presentations (Table 2). When safety planning was documented, it was facilitated by either a social worker (*n* = 4, 9.3%) or a mental health clinician (*n* = 2, 4.7%).

**TABLE 1:** Incidence of documented discussion surrounding domestic and family violence related DFV protection orders (*n* = 43).

	Count	%
DVO in place against spouse	10	23.3%
DVO in place against patient	6	14.0%
DVO was not discussed or mentioned	25	58.1%
DVO discussed yet not currently in place	2	4.7%

**TABLE 2:** Incidence of documented discussion surrounding domestic and family violence‐related safety planning.

	Count	%
DFV safety planning not undertaken	36	83.7%
Safety planning undertaken by mental health clinician	2	4.7%
Safety planning undertaken by Social Worker	4	9.3%
Patient declined to safety plan	1	2.3%

## 6 IPV Presentations and Care Provisions

Within the total sample of 43 patient presentations, paramedics were often the first to document their identification of current IPV in 32.6% (*n* = 14) of presentations. Emergency medical doctors were the first to identify and document IPV in a further 32.6% (*n* = 14) of presentations. Mental health clinicians, nurses and social workers identified current IPV in the remaining presentations. In addition, historical IPV was identified in 88.4% (*n* = 38) of presentations, highlighting the cumulative trauma experienced by many victim‐survivors. Furthermore, childhood trauma was identified in 27.9% (*n* = 12) of patient presentations, highlighting the cumulative experiences of trauma over the life course for many victims of IPV. When analysing the care provided, tasks that are linked to operational hospital policy were attended to most frequently (Table 3). For example, documenting the patient's belongings (*n* = 39, 90.7%) and skin integrity assessments (*n* = 38, 88.4%) were completed most frequently. Medical care such as the administration of medication (*n* = 31, 72.1%) or requests for specific pathology tests were also documented routinely (*n* = 29, 67.4%). Referrals to a hospital social worker were made in 53.5% (*n* = 23) of the reviewed presentations. Seventeen presentations (39.5%) received a referral to an external nongovernment IPV support service.

**TABLE 3:** Emergency department completion rates of specified care.

	Procedure/care documented (*n* = 43)		Procedure/care not documented (*n* = 43)	
	Count	%	Count	%
Pathology	29	67.4	14	32.6
Radiology	11	25.6	32	74.4
Urine specimen	18	41.9	25	58.1
ECG	10	23.3	33	76.7
Skin integrity assessment	38	88.4	5	11.6
Medication administered	31	72.1	12	27.9
Intravenous therapy	5	11.6	38	88.4
Visual observations	26	60.5	17	39.5
CALD referral	0	0	43	100.0
NGO referral	17	39.5	26	60.5
SW referral	23	53.5	20	46.5
Security referral	0	0	43	100.0
Patient belongings documented	39	90.7	4	9.3
Blood alcohol level (BAL)	8	18.6	35	81.4

## 7 Discussion

This retrospective chart review explored care provision to victim‐survivors of IPV with comorbid mental health symptoms presenting to an ED. Our findings indicate that emergency clinicians in this study (doctors, nurses, social workers and paramedics) appear to be failing to adequately respond to IPV victims primarily by not providing holistic healthcare responses that recognise the need for psychosocial risks and needs assessments. A lack of consideration of risk assessment and safety planning was notable for most of the IPV victims presenting to the ED. There is increasing recognition that risk assessment and safety planning are essential to promote the ongoing health and well‐being of patients who are experiencing IPV. These discussions and referrals are within the role and responsibilities of emergency clinicians yet appear to be rarely undertaken (Ahmad et al. 2017). Failure to adequately respond to IPV victims in terms of risk assessment and safety planning can increase the risk of further trauma and victimisation (Rogers et al. 2023). Whilst the researchers recognise that safety planning is beyond the scope of first‐responding paramedics, the lack of safety planning across contact with different clinicians during ED presentations is a gap in care provision that can put patients experiencing IPV at an increased and ongoing risk of victimisation, morbidity and mortality. Findings suggest that emergency clinicians require further IPV education, continuing professional development and organisational support to confidently assess risk, identify if there is any safety planning in place, and if not help patients in developing safety plans (Ghafournia and Healey 2022).

As noted within the existing literature (Sawyer et al. 2017), our study also suggests that paramedics may have limited knowledge and understanding of existing IPV protocols and legislation. This has been compounded by what may be the inappropriate use of the legal provisions within *The Public Health Act 2005* (*Qld*) (Office of the Queensland Parliamentary Counsel 2024). This study found paramedics routinely employ legal provisions within *The Public Health Act 2005* (*Qld*) against patients experiencing IPV who present with emotional distress, thereby pathologising victim‐survivors responses to IPV‐related trauma and forcing healthcare assessment and treatment (Office of the Queensland Parliamentary Counsel 2024). Paramedics' documented assessment of women does not appear to substantiate or meet the legislative requirement for the initiation of an EEA. The use of an EEA removes bodily autonomy from the patient, providing paramedics with the legal right to use physical and medicinal measures to transport the patient to the nearest healthcare facility for assessment (*The Public Health Act 2005* (*Qld*)). Whether the use of this legislation is appropriate or even ethical is beyond the scope of this study, however what is clear is that inappropriate use of this legislation may be a violation of patients' rights and can result in secondary victimisation, re‐traumatisation and a reluctance for the victim to seek healthcare in the future. A recommendation for further training and education for paramedics surrounding the use of the provisions within *The Public Health Act 2005* (*Qld*) in the context of IPV‐related victimisation and trauma responses is a matter of priority.

Consistent with previous work, this study supports that the ED can offer a unique opportunity to identify clinical and psychosocial support needs of victims of IPV and facilitate relevant referral pathways (García‐Moreno et al. 2015; Heron and Eisma 2021). However, the limited psychosocial needs assessments and care provisions for IPV victims observed in this study suggest that identifying IPV victims and facilitating IPV‐informed referral pathways are not prioritised in emergency settings. Evidence suggests that emergency clinicians may not have adequate knowledge, time or skills to provide holistic, trauma‐informed care to the victims of IPV (Gillespie et al. 2023). Furthermore, many support that acute physiological symptoms should take precedence over underlying trauma (Gillespie et al. 2023). Yet, the ED is not only a place for the management of acute physical injuries, but for many, it is a pathway to further support and care (Office of the Queensland Parliamentary Counsel 2024). Failure to recognise this change in operational and patient needs may have contributed to the limited referrals of patients to either social workers or community organisations working for IPV victims. This failure to holistically respond to the needs of patients experiencing IPV represents a missed opportunity to promote the patient's immediate safety and subsequent recovery. This is particularly problematic when considering that almost 90% of presentations reviewed in this study involved repeat experiences of IPV victimisation and more than one in four presentations involved cumulative experiences of adult and childhood trauma. In line with prior work, findings support the need for routine screening to maximise opportunities for IPV identification in ED settings, followed by referral pathways to psychosocial and specialist IPV support services to prevent revictimisation experiences and improve victim safety and wellbeing (Ambuel et al. 2013; Gillespie et al. 2023; Heron and Eisma 2021).

## 8 Limitations

As this research was a retrospective chart review at one regional Australian hospital, findings are limited to a small sample size. Further, findings are limited to charts where current IPV was identifiable based on healthcare clinicians' articulation of patients' victimisation experiences in clinical notes in the absence of routine screening for IPV in the study jurisdiction's ED settings. As a result, findings are unable to speak to the care experiences of IPV victims whose victimisation experiences remained unidentified. Results may not be generalisable or transferable to other regions or jurisdictions with markedly different healthcare systems, and/or differing cultural boundaries and beliefs and are limited to patients whose IPV experiences are identified by healthcare providers. Finally, this research assumes that essential information and care provided to patients should be documented in the patient's healthcare record as undocumented or poorly documented information can lead to a loss of information between care‐related contacts, which can jeopardise the patient's health and safety. As a result, identified care responses are limited to clinicians' record‐keeping and level of documentation of care.

## 9 Conclusion

Women presenting to the ED with IPV victimisation experiences and comorbid mental health symptoms have support needs that require holistic, trauma‐informed responses, addressing their physiological, mental and psychosocial well‐being. Yet, after the assessment and intervention to address a patient's physiological condition, there appears to be a lack in the provision of holistic care within the ED response to IPV victims. This was evident in the low numbers of referrals to social workers and community‐based IPV specialist services. The focus on clinical priorities and physiological care—whilst important—should not detract from the need to promote the ongoing safety and well‐being of patients affected by IPV.

## 10 Relevance to Clinical Practice

As healthcare clinicians appear to readily undertake mandatory care requirements in alignment with hospital policy, this research recommends the development and implementation of hospital‐based care policies for victim‐survivors of IPV to promote the provision of routine screening, followed by holistic, individualised, trauma‐informed care beyond addressing the patient's acute physical symptoms or presenting concerns. Such care needs to include assessments of patients' immediate and ongoing risks and support needs.

## Ethics Statement

This project received ethical approval in 2022 from The Prince Charles Hospital Human Research Ethics Committee (HREC/2022/TPCH/86965) and reciprocal ethics from the University of the Sunshine Coast and Griffith University. As patients were not directly engaged in this study and individual consent was unable to be obtained the research team was required to apply for Public Health Approval (Public Health Approval: 86965) to obtain access to patient charts. This involved a rigorous process to establish and confirm that patient privacy and confidentiality were maintained, and that the information obtained was utilised in accordance with ethical provisions.

## Conflicts of Interest

The authors declare no conflicts of interest.

## Data Availability

The data that support the findings of this study are available on request from the corresponding author. The data are not publicly available due to privacy or ethical restrictions.
